# Cervical mobility and cervical proprioception in relation to fall risk among older adults: a prospective cohort study

**DOI:** 10.1007/s41999-023-00785-y

**Published:** 2023-04-29

**Authors:** Tine Roman de Mettelinge, Patrick Desimpelaere, Dirk Cambier

**Affiliations:** 1 Department of Rehabilitation Sciences and Physiotherapy, Faculty of Medicine and Health Sciences, Ghent University, Campus UZ Gent, Corneel Heymanslaan 10, 9000 Ghent, Belgium; 2 Department of Geriatrics, AZ Maria Middelares, Ghent, Belgium

**Keywords:** Fall risk, Cervical mobility, Cervical proprioception, Fear of falling

## Abstract

**Purpose:**

Age-related decreases in cervical mobility and proprioception have previously been demonstrated. Potential associations of these deteriorations with fall risk have not been investigated so far. This study aims to compare cervical mobility and proprioception between fallers and non-fallers and prospectively assess the contribution of these parameters in fall risk identification among healthy older adults.

**Methods:**

95 community-dwelling older adults underwent a cervical screening. Active cervical range of motion (aCROM) was measured using a digital inclinometer and cervical proprioception was assessed by determining joint position error (JPE). Fear of falling was identified through the Iconographical Falls Efficacy Scale (iconFES). Falls were prospectively recorded during a 1-year follow-up period using monthly calendars. Univariate and multivariate logistic regression analyses were conducted to examine the association between these parameters and falls occurrence.

**Results:**

Baseline measurements revealed reduced cervical performance (i.e., smaller aCROM and larger JPE) among individuals who reported at least one fall during the following year (“fallers”). The multivariate logistic regression model contained eight independent variables (age, sex, walking aid, fall history, iconFES, aCROM F, aCROM E and JPE) and correctly classified 77.8% of cases.

**Conclusion:**

Although the contribution of cervical parameters to fall risk identification seems to be rather small compared to well-known (though often unmodifiable) major risk factors, further research is needed to elucidate underlying mechanisms of cervical functions in relation to falls. Second, it would be interesting to develop a targeted fall preventive cervical exercise program and assess its effectiveness in terms of falls occurrence.

## Key summary points

**Aim:**

Are age-related decreases in cervical mobility and proprioception associated with fall risk?

**Findings:**

Baseline measurements revealed reduced cervical function among individuals who reported at least one fall during the following year.

**Message:**

Further research is needed to elucidate underlying mechanisms of cervical functions in relation to falls.

## Introduction

During the past decades, getting older became the academic scope of many researchers in a wide field of sciences. The world is aging fast and older adults are the fastest growing segment of the increasing global population. The aging population poses major challenges to researchers, clinicians, society and older adults themselves. Falls are the second leading cause of unintentional injury deaths worldwide [1], and if not leading to death, falls might cause life-changing consequences for the older individual. Research in the area of fall risk detection and prevention among older adults has, therefore, boomed. Hundreds of fall risk factors have been identified, indicating that falls definitely have a complex and multifactorial etiology. A fall incidence indeed mostly occurs as a result of the interplay between intrinsic, extrinsic and situational factors [2]. The intrinsic risk factors can be divided into *functions* (such as balance, gait, muscle weakness, flexibility) needed to maintain postural control and prevent falling, and intrinsic *characteristics* (age, sex, previous falls, fear of falling, medications, pathological conditions) that may interfere with a safe and upright proactive performance of these functions [2]. When conducting fall risk detection, it is certainly important to identify some major risk factors such as age, sex and fall history. However, these risk factors are often fixed facts that are not susceptible for intervention. In terms of fall preventive initiatives, it is, therefore, crucial to determine those individual fall risk factors that are modifiable. Balance and gait impairments are considered the most important modifiable intrinsic fall risk factors [3] and have been investigated extensively. Focus of this research was often dominated by the role of the lower limbs and/or related (in)adequate central information processing. Interestingly, in his systematic review, Lin et al. concluded that there is an association between forward head posture and a detrimental alteration in limits of stability, performance-based balance, and cervical proprioception [4].

Increasing age is associated with decreased cervical ranges of motion (ROM). Every 10 years, humans loose approximately 5 degrees for active neck extension and 3 degrees for active neck flexion, lateral flexion and rotation [5, 6]. Findings concerning the potential effect of gender on active cervical range of motion (aCROM) are inconsistent. Some studies found no differences in aCROM between men and women [7-9], others showed that women generally have significantly higher aCROM than men [10, 11]. Either way, decreases in cervical ROM have strongly been associated with benign paroxysmal positional vertigo regardless of gender [12]. Furthermore, reduced neck mobility limits visual monitoring of (sudden changes in) the environment, thus providing restricted visual input to the vestibular system.

Besides an age-related decrease in cervical ROM, Lansade et al. demonstrated a significant deterioration of cervical proprioceptive abilities with increasing age [13]. Proprioception refers to the process in which information sent by afferent receptors such as peripheral muscles, capsules, ligaments and joints is processed in the central nervous system [14]. It is the ability to have both a sense of body orientation and position as well as a sense of body and limb motion [14]. Somatic afferent information from the neck, particularly the upper cervical spine, converges with vestibular and visual inputs on central nervous system nuclei involved in processing and integration of postural balance inputs [15, 16]. Abnormal cervical afferent input results in an impaired cervical position sense, which is measured as cervical joint position error (JPE) [17, 18]. Several studies showed a positive correlation between cervical JPE and age, confirming an age-related decline in cervical joint position sense [18, 19]. A study of Treleaven et al. showed significant correlations between higher cervical JPE and poorer clinical balance tests in whiplash patients, suggesting that postural control impairments may be partially explained by an altered kinesthetic sense with an increased cervical JPE [20]. Similarly, in patients with fibromyalgia syndrome associations between impaired cervical proprioception and poor balance tests have been demonstrated [21, 22]. Neck proprioception has even been illustrated as a subsystem that provides sensory input to the postural control system [23]. Although cervical statics and dynamics increasingly draw the attention of researchers in the area of balance and postural control, the potential relationship with falls has not been investigated so far.

It might be hypothesized that the age-related decrease in cervical ROM and proprioception are associated with postural control disturbances, resulting in an increased fall risk. This study aims (i) to compare cervical ROM and proprioception between fallers and non-fallers and (ii) to prospectively assess the contribution of these parameters in fall risk identification among healthy older adults.

## Materials and methods

### Ethics statement

The Ethical Committee of the Ghent University and the Ghent University Hospital gave approval too this study and all participants signed an informed consent.

### Participants

Participants were recruited through online advertising, flyer distribution and by word of mouth. Inclusion criteria were: (i) being 65 years or older, (ii) living independently, and (iii) being able to walk independently with or without a walking aid. People were excluded if they presented with (i) severe musculoskeletal (e.g., amputations, foot deformities, and major rheumatic conditions in the lower extremities) or neurological disorders (stroke, Parkinson’s disease, etc.) that might affect balance and/or gait, (ii) severe cognitive disorders, (iii) notable cervical pain (VAS > 8/10), and (iv) dizziness due to underlying disorders such as vestibular dysfunction or whiplash associated disorders.

### Personal and medical history, fear of falling

Socio-demographic data, medical history and fall history were recorded by means of a questionnaire. Fear of falling was assessed with the Dutch version of the Iconographical Falls Efficacy Scale (iconFES, 30 items) mobile application [24]. The iconFES uses pictures to provide clear, unambiguous contexts [24]. Each item is scored on a 4-point scale (1 = not at all concerned to 4 = very concerned), along with facial expression icons to assess the level of concern [24]. Total score ranges from 30 (corresponding to “no concern”) to 120 (corresponding to “extremely concerned about falling”) during the performance of the specific activities suggested by the questionnaire [24].

### Physical measurements

Cervical mobility was measured by the Acumar® Single Digital Inclinometer (Acumar, Lafayette Instrument, Lagatette, IN, USA). Digital inclinometers have proven to be valid instruments for assessing CROM [25]. For aCROM measurements of flexion, extension and lateral flexion, participants were seated in a chair with backrest, with hips and knees flexed at 90°, feet on the ground, forearms and hands resting on the upper legs and head straight ahead. aCROM of rotation was measured while the participant was placed in a supine position on a standard examination table with the shoulders on the table, arms alongside the body, legs bended and head facing the ceiling. Subsequently, maximal active cervical flexion (F), extension (E), right and left lateral flexion (RLF and LLF), and right and left rotation (RR and LR) were executed. Motions in the same plane were performed alternately (e.g., in the sagittal plane: F, then E, then F, etc.). Participants were instructed to perform their maximal cervical motion at a comfortable, self-selected pace. All motions were repeated 3 times. The mean value of three trials for each movement was retained for statistical analyses (degrees).

To determine joint position error (JPE), the active movement angle reproduction test was used. Participants were asked to sit upright on a chair with a backrest, blindfolded, in a neutral but comfortable head position (NHP) with hips and knees flexed at approximately 90° at a fixed distance of 90 cm to a wall, wearing a headlight laser pointer on the head [26]. Participants were then instructed to slowly perform a full active head rotation to one side and subsequently return back to the NHP. Intra- and inter-rater reliabilities of this NHP test have proven to be good to very good [27]. To optimize stability and reliability of outcome measures, 10 trials for each movement were registered [28]. First, 10 trials to the left were executed. After a few minutes, participants performed 10 trials to the right. Before each trial, the examiner manually repositioned the subject’s head to the initial starting position. During the test procedure, no tactile or verbal feedback was given about the participant’s performance. Participants were asked to verbally report when they perceived that NHP had been attained. The endpoint after each rotation was marked and indicated the global error related to the zero point, i.e., NHP. Mean cervical joint position error, in degrees, was calculated as (right JPE + left JPE)/2.

After completion of an extensive training period for aCROM and JPE testing, two examiners independently administered these physical measurements. In order to avoid major effects of diurnal variation in cervical mobility and proprioception, physical measurements were planned at least 2 h after the subject woke up.

### Falls follow-up

After baseline measurements, falls were monitored during 1 year. Participants were provided with fall calendars on postcard format. They were asked to record fall events and monthly send these postcards to the Department of Medicine and Health Sciences. A fall was defined as “an unexpected event in which the person comes to rest on the ground, floor, or lower level” [29]. If a fall occurred, participants were contacted and asked about the circumstances and eventual fall-related injuries. Participants were also contacted by phone if a monthly postcard was not received. A participant who experienced at least one fall during the follow-up period was categorized as “faller”. If no fall event occurred during follow-up, the participant was categorized as “non-faller”.

### Statistical analyses

Besides descriptive statistics to represent population characteristics, Independent Samples *T* Test (if data were normally distributed), Mann–Whitney *U* test (if data were not normally distributed) and Chi-squared test were used to assess differences in age, sex and cervical performances between fallers and non-fallers.

Univariate and multivariate logistic regression models were applied to examine the odds of fall events (during a 1-year follow-up) as a function of neck abilities (mobility and proprioceptive function) and some potential well-known personal fall risk factors. The independent variables were age, gender, use of walking aids, fall history, fear of falling, cervical mobility and cervical proprioception. To rule out multicollinearity, correlation matrices and Pearson correlation coefficients for the aCROM data were calculated.

All statistical analyses were performed using SPSS for Windows, Version 28.0 (IBM SPSS, Inc., Chicago, IL). Statistical significance was assumed at *p* < 0.05.

## Results

98 healthy older adults volunteered to participate. Three of them did not meet inclusion criteria: one man had a clubfoot, another man was diagnosed with cancer and a vestibular disorder and a woman suffered a whiplash injury. As such, 95 older adults were enrolled in this study. Four (out of 95) participants withdrew during the follow-up period. One case was identified as outlier during multivariate logistic regression analysis (ZResid-3.3) and was, therefore, excluded in statistical analyses.

Mean age of the cohort is 75.7 ± 7.5 (range 65–93) and approximately 2 out of 3 participants were female. 43.2% of this cohort experienced at least one fall during the past year (“retrospective faller”) and a comparable proportion (38.9%) reported at least one fall during the 1-year follow-up period (“prospective faller”). Table 1 summarizes study population characteristics.

**Table 1:** Study population characteristics

Characteristics	Values (*N* = 95)
Age (years), mean ± SD	75.7 ± 7.5
BMI (kg/m^2^), mean ± SD	26.1 ± 4.0
Female, *n* (%)	62 (65.3)
Walking aid, *n* (%)	22 (23.2)
Retrospective faller, *n* (%)	41 (43.2)
Prospective faller, *n* (%)	37 (38.9)
iconFES, mean ± SD	53.0 ± 19.6

*iconFES* iconographical falls efficacy scale

In Table 2, comparison of population characteristics and cervical properties between prospective fallers and non-fallers are presented. Participants who reported at least one fall event during the 1-year follow-up period (“fallers”), used walking aids significantly more often and had a positive fall history (“retrospective fallers”). They had also a higher score on iconFES, indicating that they were significantly more concerned about falling compared to the non-fallers. Except for flexion, neck mobility in all movement directions was lower in fallers than in non-fallers. However, only aCROM for extension was statistically significant. JPE was higher in fallers compared to non-fallers, albeit not statistically significant.

**Table 2:** Comparison of characteristics between prospective fallers and non-fallers

	Faller (*n* = 37)	Non-faller (*n* = 53)	*p*-value	Odds ratio (95% CI)
Female, *n* (%)	23 (39)	36 (61)	0.654	0.78 (0.32–1.87)
Age (years)	77.4 ± 8.3	74.4 ± 6.6	0.074^^^	1.06 (1.00–1.12)^^^
Walking aid, *n* (%)	13 (68.4)	6 (31.6)	0.009*	4.24 (1.43–12.56)*
Retrospective faller, *n* (%)	25 (65.8)	13 (34.2)	< 0.001**	6.41 (2.53–16.25)**
iconFES, mean ± SD	60.4 ± 21.2	45.9 ± 15.1	< 0.001**	1.04 (1.01–1.06)**
aCROM (°), mean ± SD				
F	49.8 ± 12.8	49.0 ± 10.44	0.749	1.01 (0.97–1.04)
E	42.6 ± 12.9	49.5 ± 10.9	0.007*	0.95 (0.91–0.99)*
RLF	23.8 ± 8.1	27.3 ± 7.7	0.041*	0.94 (0.89–1.00)*
LLF	26.4 ± 7.6	29.0 ± 7.6	0.114	0.96 (0.90–1.01)
LF L + R	50.2 ± 15.1	56.3 ± 14.6	0.058^^^	0.97 (0.94–1.00)^^^
RR	51.3 ± 15.6	56.0 ± 14.6	0.178	0.98 (0.95–1.01)
LR	52.5 ± 14.7	55.5 ± 15.3	0.377	0.99 (0.96–1.01)
R L + R	103.8 ± 28.9	111.6 ± 28.6	0.243	0.99 (0.98–1.01)
JPE (°), mean ± SD				
JPE-mean	4.8 ± 2.3	4.1 ± 1.6	0.093^^^	1.22 (0.98–1.53)^^^

Odds ratios for univariate risk factors of experiencing at least one fall event during 1-year follow-up

*iconFES* iconographical falls efficacy scale, *aCROM* active cervical range of motion, *SD* standard deviation, *F* flexion, *E* extension, *RLF* right lateral flexion, *LLF* left lateral flexion, *RR* right rotation, *LR* left rotation, *JPE* joint position error, *JPE*-*mean* mean joint position error for left and right rotation to neutral head position

^^^*p* < 0.1, *p < 0.05, **p < 0.001

The characteristics were then entered in separate single logistic regression models. The last column of Table 2 shows the Odds Ratios for the univariate risk factors of these logistic regression models.

Significantly moderate to strong correlation coefficients were found between aCROM E and aCROM values for lateral flexion (Pearson correlation coefficient *r* = 0.456–0.495) and rotation (*r* = 0.547–0.592), but not between aCROM E and aCROM F. To avoid multicollinearity issues, it was, therefore, opted to only include aCROM for extension as an independent variable for cervical mobility in multivariate logistic regression analysis (Table 3). CROM F was not included in multivariate logistic regression since it was not a significant univariate fall risk factor, as shown in Table 2.

**Table 3:** Multivariate logistic regression predicting likelihood of reporting at least one fall event during 1-year follow-up

	B	S.E	Wald	*df*	*p*	Odds ratio (95% CI)
Sex	− 1.023	0.637	2.583	1	0.108	0.36 (0.10–1.25)
Age	− 0.059	0.048	1.537	1	0.215	0.94 (0.86–1.04)
Walking aid	0.366	0.792	0.213	1	0.644	1.44 (0.31–6.80)
Retrospective faller	1.703	0.597	8.132	1	0.004*	5.49 (1.70–17.70)
iconFES	0.042	0.017	6.473	1	0.011*	1.04 (1.01–1.08)
aCROM						
E	− 0.017	0.026	0.423	1	0.515	0.98 (0.94–1.03)
JPE						
JPE-mean	0.206	0.138	2.236	1	0.135	1.23 (0.94–1.61)
Constant	1.606	3.719	0.187	1	0.666	4.99

*iconFES* iconographical falls efficacy scale, *aCROM* active cervical range of motion, *E* extension, *JPE* joint position error, *JPE*-mean (JPE-R + JPE-L)/2

*p < 0.05

Logistic regression was performed to assess the impact of a number of factors on likelihood that respondents would report a fall event during the 1-year follow-up period. The model contained eight independent variables (age, gender, walking aid, retrospective faller, iconFES, aCROM E and JPE-mean). The full model containing all predictors was statistically significant, *χ*^2^ (7, *N* = 90) = 29.684,* p* < 0.001, indicating that the model was able to distinguish between respondents who did and did not report a fall event. The model correctly classified 77.8% of cases with a sensitivity of 67.6% and a specificity of 84.9%. As shown in Table 3, only two of the independent variables made a unique statistically significant contribution to the model (retrospective faller and iconFES). The strongest predictor of reporting a fall event was retrospective faller, recording an odds ratio of 5.49.

## Discussion

Hundreds of fall risk factors among older adults have been identified in the past. However, little attention is paid to the potential role of the central axis of the body (i.e., trunk and neck) in this context. This study aimed to compare cervical abilities between fallers and non-fallers and to assess the contribution of these parameters in fall risk identification among healthy older adults.

Our results confirmed the hypothesis that neck mobility is smaller in fallers compared to non-fallers. Interestingly, this was not the case for cervical flexion with mean ROM that was even slightly larger in fallers. ACROM for right lateral flexion was significantly smaller in fallers compared to non-fallers, whereas aCROM for left lateral flexion did not reach significance level. It might be interesting to assess potential relationships between the predominance of motor function laterality and ipsilateral aCROM, in an attempt to explain this finding.

Univariate analyses showed that older individuals who suffered at least one fall during the 1-year follow-up period were more likely to report falls during the previous year, walk with mobility aids, have more fear of falling, and present with a lower aCROM for extension and right lateral flexion. ACROM for summed lateral flexion and mean JPE appeared to be borderline univariate fall risk factors. Our results are in line with the main fall risk factors that have clearly been identified in the past: a positive fall history, using walking aids and fear of falling [30]. Neck proprioception has previously been identified as a subsystem that provides sensory input to the postural control system [23]. As such, disturbances to the afferent input from the cervical region (e.g., in those with neck pain) may be a possible cause of symptoms such as dizziness, unsteadiness and visual disturbances, as well as signs of altered postural stability, cervical proprioception and head and eye movement control [23]. Although cervical statics and dynamics increasingly draw the attention of researchers in the area of balance and postural control, the potential relationship with falls has not been investigated so far.

Age, gender, walking aid, retrospective faller, iconFES, aCROM E and JPE-mean were entered in a multivariate logistic regression model. The major factors influencing whether an older individual will report a fall incidence are reporting falls in the previous year and having a high level of fear of falling (high iconFES score). This study showed that respondents who experienced one or more falls during the past year are over 5 times more likely to report a fall event during the following year than those who had a negative fall history, controlling for all other factors in the model.

The other variables did not contribute significantly to the model. The contribution of cervical parameters to fall risk identification seems to be rather small compared to well-known major fall risk factors. This is not surprising since it is very plausible that latter powerful variables (i.e., fall history and fear of falling) have canceled out the impact of other risk factors such as cervical abilities. However, it is not an option to generate a model that only includes cervical parameters as this would be little meaningful. The universally accepted major fall risk factors (age, fall history, fear of falling, etc.) cannot be ignored in any fall risk detection strategy. In terms of fall prevention strategies, however, identifying the “smaller” and person-specific fall risk factors might be valuable. Actually, the smaller fall risk factors often seem to be partly or fully reversible. Contrary to major fall risk factors like age and fall history which are irrevocable characteristics, smaller fall risk factors like reduced cervical mobility and proprioception might be modifiable through tailored exercise programs.

The findings of this study suggest a cause–effect relationship in terms of reduced cervical proprioception and mobility leading to increased fall risk. However, the reverse reasoning might be interesting too. In an attempt to prevent fall events, fallers may take a more cautious movement pattern by locking their head on their trunk. Although the underlying mechanisms are still unclear, there is a growing awareness and interest in the relationship of neck kinematics and falls. Kuo et al. recently investigated how neck musculature is used to avoid head impact during falls in older adults [31]. They suggest that understanding these strategies might lead to the development of therapeutic exercise programs pursuing safe landing during falls. Similarly, we believe that gaining insight in the impact of cervical abilities on fall occurrence might contribute to tailored fall preventive (cervical) exercise therapy. Some preliminary research suggests that proprioceptive training of a properly working system might have little surplus value in terms of improvement of cervical performance [32]. Others found promising results of oculomotor and gaze stability exercises in terms of improving balance impairment [33] and reducing fall risk [34].

The current study is subject to several methodological considerations. Based on the finding of Swait et al. that at least 6 trials of cervical JPE test were needed to obtain good test–retest reliability [28], we performed 10 trials for left repositioning movement and 10 trials for right repositioning movement. This large number of repetitions might, however, have induced some fatigability of cervical muscles, potentially influencing JPE test results. Second, physical assessment was administered by 2 examiners. Although both examiners completed an extensive training period for aCROM and JPE testing, this might have influenced inter-rater reliability. Thirdly, a recall bias might have occurred when the older participants were asked about their fall history. To avoid reporting bias using the fall calendars, participants were contacted by phone if a monthly postcard was not received. Finally, an a priori sample size (power) calculation was not performed. Despite these weaknesses, this is the first study prospectively investigating potential associations of cervical mobility and proprioception with fall risk among healthy older adults, which can be considered the major strength of this study.

## Conclusions

Baseline measurements revealed reduced cervical performance among individuals who reported at least one fall during the following year. Although the contribution of cervical parameters to fall risk identification seems to be rather small compared to well-known (though often unmodifiable) major risk factors, further research is needed to elucidate underlying mechanisms of cervical abilities in relation to falls. It might, therefore, be valuable to include balance performance in future research. Given the inconsistent preliminary findings in this context, large studies are required to formulate clear and unanimous conclusions. Finally, it would be interesting to develop a targeted fall preventive cervical exercise program and assess its effectiveness in terms of falls occurrence.

## Data Availability

Data are available at Ghent University, Faculty of Medicine and Health Sciences, Dpt. Rehabilitation Sciences. Interested parties are advised to contact the corresponding author (Tine.RomandeMettelinge@Ugent.be).
